# Dynamics and Control of the Central Carbon Metabolism in Hepatoma Cells

**DOI:** 10.1186/1752-0509-4-54

**Published:** 2010-04-28

**Authors:** Klaus Maier, Ute Hofmann, Matthias Reuss, Klaus Mauch

**Affiliations:** 1 Institute of Biochemical Engineering, University of Stuttgart, Allmandring 31, 70569 Stuttgart, Germany; 2 Dr Margarete Fischer-Bosch Institute of Clinical Pharmacology, Stuttgart and University of Tuebingen, Auerbachstrasse 112, 70376 Stuttgart, Germany; 3 Insilico Biotechnology AG, Nobelstrasse 15, 70569 Stuttgart, Germany

## Abstract

**Background:**

The liver plays a major role in metabolism and performs a number of vital functions in the body. Therefore, the determination of hepatic metabolite dynamics and the analysis of the control of the respective biochemical pathways are of great pharmacological and medical importance. Extra- and intracellular time-series data from stimulus-response experiments are gaining in importance in the identification of *in vivo *metabolite dynamics, while dynamic network models are excellent tools for analyzing complex metabolic control patterns. This is the first study that has been undertaken on the data-driven identification of a dynamic liver central carbon metabolism model and its application in the analysis of the distribution of metabolic control in hepatoma cells.

**Results:**

Dynamic metabolite data were collected from HepG2 cells after they had been deprived of extracellular glucose. The concentration of 25 extra- and intracellular intermediates was quantified using HPLC, LC-MS-MS, and GC-MS. The *in silico *metabolite dynamics were in accordance with the experimental data. The central carbon metabolism of hepatomas was further analyzed with a particular focus on the control of metabolite concentrations and metabolic fluxes. It was observed that the enzyme glucose-6-phosphate dehydrogenase exerted substantial negative control over the glycolytic flux, whereas oxidative phosphorylation had a significant positive control. The control over the rate of NADPH consumption was found to be shared between the NADPH-demand itself (0.65) and the NADPH supply (0.38).

**Conclusions:**

Based on time-series data, a dynamic central carbon metabolism model was developed for the investigation of new and complex metabolic control patterns in hepatoma cells. The control patterns found support the hypotheses that the glucose-6-phosphate dehydrogenase and the Warburg effect are promising targets for tumor treatment. The systems-oriented identification of metabolite dynamics is a first step towards the genome-based assessment of potential risks posed by nutrients and drugs.

## Background

Dynamic network models of the hepatic metabolism enable quantitative systems-level analyses of (i) detailed metabolic control patterns, (ii) metabolic implications in liver cancer, and (iii) metabolic processes such as detoxification. Moreover, systems-oriented analyses of the dynamics and control of the central carbon metabolism in the liver are an important step on the avenue towards the personalized prognosis of drug actions and/or long-term effects. This will eventually lead to a reduction in potential side effects and healthcare costs as well as enabling quick, rational decisions to be made in the course of expensive drug discovery processes. However, due to the limitations of wet and dry lab procedures [1,2], model-based analyses of the liver metabolism have so far mainly focused on the identification of metabolic fluxes [3-7] and the coarse-grained quantification of the control of metabolic sub-networks [8-11]. It is worth noting that the analysis of metabolic control patterns using dynamic network models enables a more detailed interpretation of the hepatic control distribution than could be achieved with top-down approaches. In the context of oxidative phosphorylation and the dynamic interplay of catabolism and anabolism, the cofactors NAD(H), NADP(H), ATP/ADP/AMP need to be taken into account by mass balances when analyzing the systems-level effect of the energy metabolism. However, for identifying network models time-series of cofactor concentrations have until now mainly been used in external approximation functions [12-14] rather than for predicting the effect of cofactor concentrations on metabolic fluxes and intermediate concentrations.

Several metabolic functions and processes are constantly and concurrently maintained in the liver, which is a complex organ performing a plethora of vital functions [15]. These functions include the biosynthesis of cholesterol and bile acids, the bilirubin-, porphyrin-, and carbohydrate metabolisms as well as the detoxification of xenobiotics. The detoxification metabolism, i.e. the phase I and phase II degradation of exo- and endogenous substances, is directly linked with the central carbon metabolism, as it relies on the adequate supply of precursors such as NADPH and UDP-glucuronide. Moreover, glucose homeostasis is another liver-specific task of major pharmaceutical and medical importance, and should not be analyzed without taking into account the central carbon metabolism [16]. Liver cells have an important role in the metabolism of lipids. In the fed state, fatty acids are actively synthesized, esterified, and secreted into the bloodstream along with very-low-density lipoproteins (VLDL). During starvation fatty acids are degraded and ketone bodies are released [16]. The human hepatoma-derived cell line HepG2 has retained several characteristic liver-specific metabolic functions, and is therefore regarded as an excellent means for examining the liver metabolism [17-19]. Furthermore, HepG2 cells were derived from a hepatoblastoma carcinoma, and therefore facilitate the investigation of the effects of tumors on the hepatic metabolism.

When building a dynamic model, the enzyme kinetics can either be deduced from non-physiological *in vitro *measurements or from intracellular metabolite time-series data. Teusink et al. reconstructed a dynamic model of yeast glycolysis based on *in vitro *kinetics; the authors observed substantial differences between model-predicted and experimentally determined *in vivo *metabolite levels [20]. Therefore, Chassagnole et al. and Nikerel et al. advocate the use of *in vivo *metabolite time-series data for the identification of intracellular enzyme kinetics [21,22]. This creates the need for sophisticated procedures for (i) the quenching of the metabolism, (ii) the extraction of intracellular metabolites, and (iii) the absolute quantification of intermediate concentrations [23]. Hofmann et al. succeeded in providing such procedures for quantifying central carbon metabolites in HepG2 cells [6]. Luo et al. used liquid-chromatography mass spectrometry to quantify the intracellular metabolites in glycolysis, the pentose-phosphate pathway, and the tricarboxylic cycle in *Escherichia coli *[24]. Schaub and Reuss investigated the *in vivo *dynamics of glycolytic intermediates in *Escherichia coli *and showed the importance of growth rate-dependent metabolome analysis [25]. So far, transient metabolite data have mainly been used for deducing the kinetic parameters in dynamic models of metabolic pathways in prokaryotes and yeast. Rizzi et al. reconstructed a dynamic model of glycolysis and the tricarboxylic acid cycle in *Saccharomyces cerevisiae *[12]. Enzyme kinetics were modeled with mechanistic rate equations and the kinetic parameters were identified in stimulus response experiments [26]. Chassagnole et al. used mechanistic rate equations and metabolite time-series data to build a dynamic model of the central carbon metabolism in *Escherichia coli *[21]. Kresnowati et al. exemplified the parameterization of a dynamic model based on linlog kinetics from artificial metabolite time-series data [27]. Magnus et al. applied linlog kinetics and intracellular intermediate measurements to model metabolite dynamics in the valine/leucine synthesis pathway in *Corynebacterium glutamicum *[13].

The analysis of metabolic control provides a mathematical framework for quantifying the responses of fluxes and intermediate concentrations to changes in internal and external parameters such as nutrient and drug concentrations at the systems level [28-31]. Control coefficients determine the amount of control exerted on a flux or concentration at any step in a particular pathway. The control of the central carbon metabolism in the liver has been studied in cells isolated from fed rats using top-down methods [8]. The reactions of the cell were grouped into nine blocks that were linked to each other by five intermediates. The control pattern observed quantified how the sub-systems interacted with one another. Elasticities were determined experimentally using the multiple modulation approach. Subsequently, Ainscow and Brand used the elasticities and control coefficients to evaluate the mutual importance of internal regulatory pathways [32]. In order to quantify the effect of a distinct block on the steady state flux through another block, the flux control coefficients were divided into partial flux control coefficients. Internal response coefficients were determined in order to assess the blocks that are most important in counteracting an increase in intermediate. The authors also investigated the effects of hormonal stimuli on rat hepatocytes by comparing the fractional changes in the fluxes and intermediate levels using the previously determined kinetics [33]. Soboll et al. applied top-down methods to analyze the control distribution in oxidative phosphorylation, gluconeogenesis, ureagenesis, and ATP turnover in isolated perfused rat liver [34]. The authors observed different control patterns for the active and inactive states. By titration with a specific phosphorylase inhibitor (CP-91149), Aiston et al. found that the phosphorylase enzyme had substantial control over glycogen synthesis in rat hepatocytes [35]. The authors concluded that the phosphorylase enzyme is a promising target for controlling hyperglycaemia in type-2 diabetes, both in the absorptive and post-absorptive states. Groen et al. applied the double modulation method to determine the elasticities and flux control coefficients of the enzymes involved in the gluconeogenetic pathway in rat hepatocytes [36]. The largest flux control coefficient was found for pyruvate carboxylase, regardless of whether glucagon was administered or not. In order to determine flux control coefficients as a function of the extracellular glucose level in rat hepatocytes, Meléndez-Hevia et al. set up a model that comprised the first three glycolytic enzymes [10]. At physiological glucose concentrations, glucokinase exerted the greatest control over the glycolytic flux. These results were similar to experimental observations performed with rat liver homogenates. Sabate et al. reconstructed from literature data a dynamic model of the pentose-phosphate pathway in fasted rat hepatocytes [37]. A sensitivity analysis revealed that the metabolic fluxes were mainly regulated by the glucose-6-phosphate dehydrogenase and transketolase enzymes.

The objective of this study is to provide systems-level analyses of the dynamics and control of the central carbon metabolism in hepatoma cells. Transient extra- and intracellular intermediate concentrations were experimentally observed in HepG2 cells in a stimulus response experiment. The experimental data were then used to parameterize a dynamic network model of the hepatic central carbon metabolism. The reaction kinetics were approximated using canonical linlog kinetics. This approach yields a good approximation quality while only requiring the determination of comparatively few parameters [22,38,39]. Systems-level effects were deduced from the analysis of metabolic control. In contrast to previous analyses, the control patterns quantified the mutual influences of individual enzymes rather than describing how the sub-systems interacted with each other. In other words, using a dynamic network model allows for a more detailed investigation of the underlying control principles. Internal regulatory pathways were further quantified by breaking up flux control coefficients into partial flux control coefficients. Internal response coefficients were investigated to assess system responses to changes in intermediates.

## Results and Discussion

**Figure 1:**
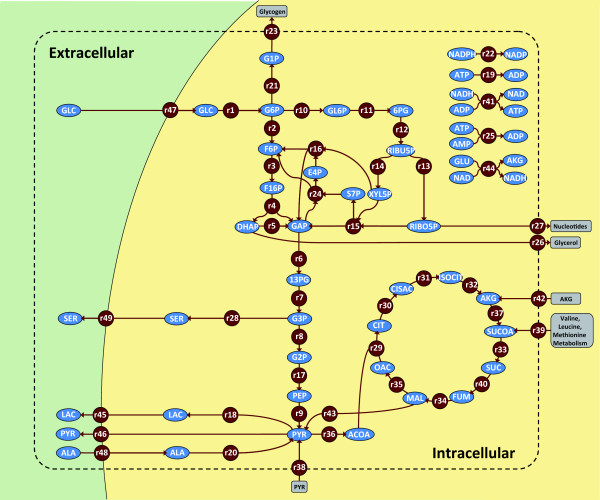
**Metabolic network model**. Extra- and intracellular metabolites: blue ellipses. Enzymatic reactions and transportation steps: red circles. Non-balanced compounds: within gray, round-edged rectangles. Directions of arrows reflect the direction of the steady state fluxes. System boundary: dashed line. Extra- and intracellular space: white and gray. Some links were omitted for reasons of clarity (cf. Table 1 for the complete reaction stoichiometry)

In the present study, a stimulus response experiment was performed with HepG2 cells. After growing HepG2 cells on a glucose-containing medium, they were incubated with fresh medium for two hours and then exposed to a medium lacking glucose. Metabolite time-series data were determined and used to parameterize a dynamic network model of the central carbon metabolism. The model takes into account 49 reactions (including 5 transportation steps) that convert 45 balanced compounds (40 intracellular and 5 extracellular metabolites). The metabolic network is depicted in figure 1 and the reaction stoichiometry is listed in Table 1 (see also the model reconstruction in a subsection of the Methods section).

**Table 1:** Reaction stoichiometry of the dynamic network model.

		Stoichiometry	
		
Reaction number	Reaction name	Substrates	Products
r1	glucokinase	GLC_in _+ ATP_in_	G6P_in _+ ADP_in _+ H_nb_
r2	glucose-6-phosphate isomerase	G6P_in_	F6P_in_
r3	phosphofructokinase	F6P_in _+ ATP_in_	F16P_in _+ ADP_in _+ H_nb_
r4	fructose-bisphosphate aldolase	F16P_in_	DHAP_in _+ GAP_in_
r5	triose-phosphate isomerase	DHAP_in_	GAP_in_
r6	glyceraldehyde-3-phosphate dehydrogenase	GAP_in _+ P_nb _+ NAD_in_	13PG_in _+ NADH_in _+ H_nb_
r7	phosphoglycerate kinase	13PG_in _+ ADP_in _+ H_nb_	G3P_in _+ ATP_in_
r8	phosphoglycerate mutase	G3P_in_	G2P_in_
r9	pyruvate kinase	PEP_in _+ ADP_in _+ H_nb_	PYR_in _+ ATP_in_
r10	glucose-6-phosphate dehydrogenase	G6P_in _+ NADP_in_	GL6P_in _+ NADPH_in _+ H_nb_
r11	6-phosphogluconolactonase	GL6P_in _+ H2O_nb_	6GP_in _+ H_nb_
r12	phosphogluconate dehydrogenase	6GP_in _+ NADP_in_	RIBU5P_in _+ CO2_nb _+ NADPH_in_
r13	ribose-5-phosphate isomerase	RIBU5P_in_	RIBO5P_in_
r14	ribulose-phosphate 3-epimerase	RIBU5P_in_	XYL5P_in_
r15	transketolase	RIBO5P_in _+ XYL5P_in_	GAP_in _+ S7P_in_
r16	transketolase	XYL5P_in _+ E4P_in_	GAP_in _+ F6P_in_
r17	phosphopyruvate hydratase	G2P_in_	PEP_in _+ H2O_nb _+ H_nb_
r18	lactate dehydrogenase	PYR_in _+ NADH_in _+ H_nb_	LAC_in _+ NAD_in_
r19	adenosinetriphosphatase	ATP_in _+ H2O_nb_	ADP_in _+ P_nb _+ H_nb_
r20	alanine transaminase	ALA_in _+ AKG_in_	PYR_in _+ GLU_in_
r21	phosphoglucomutase	G6P_in_	G1P_in_
r22	nadph consumption	NADPH_in _+ A_nb_	NADP_in _+ AH_nb_
r23	glycogen synthesis	UTP_nb _+ G1P_in_	UDP_nb _+ GLYG_nb _+ H_nb _+ PP_nb_
r24	transaldolase	S7P_in _+ GAP_in_	F6P_in _+ E4P_in_
r25	adenylate kinase	ATP_in _+ AMP_in_	2·ADP_in_
r26	glycerol synthesis	DHAP_in_	GLYCEROL_nb_
r27	nucleotide synthesis	RIBO5P_in _+ ATP_in_	PRPP_in _+ AMP_in _+ H_nb_
r28	serine synthesis	G3P_in _+ NAD_in _+ GLU_in _+ H2O_nb_	SER_in _+ P_nb _+ NADH_in _+ 2·H_nb _+ AKG_in_
r29	citrate synthase	OAC_in _+ ACCOA_in _+ H2O_nb_	CIT_in _+ COA_in _+ H_nb_
r30	aconitate hydratase	CIT_in_	CISAC_in _+ H2O_nb_
r31	aconitate hydratase	CISAC_in _+ H2O_nb_	ISOCIT_in_
r32	isocitrate dehydrogenase	ISOCIT_in _+ NAD_in_	AKG_in _+ CO2_nb _+ NADH_in_
r33	succinate-CoA ligase	SUCCOA_in _+ P_nb _+ ADP_in_	SUC_in _+ COA_nb _+ ATP_in_
r34	fumarate hydratase	FUM_in _+ H2O_nb_	MAL_in_
r35	malate dehydrogenase	MAL_in _+ NAD_in_	OAC_in _+ NADH_in _+ H_nb_
r36	pyruvate dehydrogenase complex	PYR_in _+ NAD_in _+ COA_nb_	CO2_nb _+ NADH_in _+ ACCOA_in_
r37	alpha-ketoglutarate dehydrogenase complex	AKG_in _+ NAD_in _+ COA_nb_	SUCCOA_in _+ CO2_nb _+ NADH_in_
r38	pyruvate synthesis	PYR_nb_	PYR_in_
r39	valine leucine isoleucine metabolism	ISOVALMET_nb_	SUCCOA_in_
r40	succinate dehydrogenase	SUC_in _+ 0.6·NAD_in _+0.2·O2_nb_	FUM_in _+ 0.6·NADH_in _+ 0.6·H_nb _+ 0.4·H2O_in_
r41	oxidative phosphorylation	NADH_in _+ 0.5·O2_nb _+ 3.5·H_nb _+ 2.5·ADP_in _+ 2.5·P_nb_	NAD_in _+ 3.5·H2O_nb _+ 2.5·ATP_in_
r42	alpha-ketoglutarate synthesis	AKG_nb_	AKG_in_
r43	malic enzyme	MAL_in _+ NADP_in_	PYR_in _+ CO2_nb _+ NADPH_in_
r44	glutamate dehydrogenase	GLU_in _+ H2O_nb _+ NAD_in_	AKG_in _+ NH4_nb _+ NADH_in _+ H_nb_
r45	lactate transport	LAC_in_	LAC_ex_
r46	pyruvate transport	PYR_in_	PYR_ex_
r47	glucose transport	GLC_in_	GLC_ex_
r48	alanine transport	ALA_ex_	ALA_in_
r49	serine transport	SER_in_	SER_in_

The subscripts 'ex', 'in', and 'nb' denote extracellular, intracellular, and non-balanced metabolites, respectively.

The Phosphoenolpyruvate carboxykinase enzyme was found to be inactive in the reference state [7], and, thus, it was not included in the dynamic network model.

The following paragraphs will focus on the concordance of the model simulations with the experimental data and on the application of the model for quantifying and interpreting the distribution of metabolic control.

### *In Vivo *and *In Silico *Metabolite Dynamics

**Figure 2:**
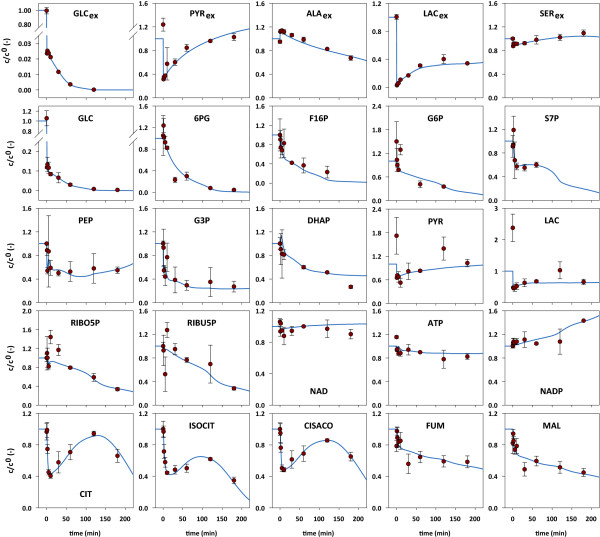
**Extracellular and intracellular metabolite dynamics**. The subscripts 'in' and 'ex' denote intracellular and extracellular metabolites, respectively. The concentration values were normalized with respect to their reference values, i.e. the concentrations directly before the stimulus. The error bars indicate standard deviations of the experimental data. To perturb the central metabolism, the glucose-containing culture medium was exchanged with glucose-free medium. By consequence, the extracellular glucose level dropped, and this stimulated significant intracellular metabolite dynamics.

A total of 25 metabolite time courses were experimentally determined, of which 5 corresponded to extracellular metabolites and 20 to intracellular metabolites. The experimental data and the corresponding model simulations are summarized in Figure 2. *In vivo *and *in silico *data were normalized with respect to the estimated reference values. It is worth noting that the perturbation triggered significant changes in the metabolite levels, and these changes provided important information about the underlying network dynamics.

After exchanging the glucose-containing culture medium with the glucose-free medium, the extracellular glucose level dropped drastically. The remaining extracellular glucose was consumed by the cells within a period of 120 min. The extracellular pyruvate and lactate levels also dropped considerably because of the medium exchange, but started to accumulate again. At the end of the experiment, i.e. after 180 min, the pyruvate values were even slightly higher than the estimated reference level. Lactate did not reach 50% of its reference value, which was the result of a decreasing lactate secretion rate. The initial efflux rate was twenty times higher for lactate than for pyruvate. This means that, in absolute terms, still more lactate than pyruvate was produced during the experiment. Extracellular alanine was consumed throughout the experiment, while extracellular serine accumulated. It is worth noting that besides the lack of glucose, the system was also perturbed as a result of the changes occurring in the extracellular pyruvate, lactate, alanine, and serine levels.

In accordance with the extracellular glucose levels, the intracellular glucose pool also decreased steeply. HepG2 cells have high GLUT2 transporter activities [40]. The GLUT2 transporter, which has a large K_m _value, facilitates the diffusion of glucose into or out of the cells [41]. It can therefore be assumed that the steep decrease in the intracellular glucose pool was the result of the diffusion of intracellular glucose into the extracellular space. Consistently, the model simulations showed that the flux of glucose uptake was inversed immediately after the stimulus occurred. The intracellular glucose concentration further decreased and eventually converged to zero. All other glycolytic metabolite levels except for phosphoenolpyruvate and pyruvate decreased sharply immediately after the stimulus and continued to gradually decrease thereafter.

In the first 10 min of the experiment, the model simulations showed decreasing ribose-5-phosphate and ribulose-5-phosphate levels, followed by an increase, and then another decrease. Some discrepancy between the initial experimental data points and the simulations was observed for both metabolites, which could be an indication of a damped oscillation with rather high amplitude.

The first TCA cycle intermediate pools, i.e. citrate, cis-aconitate, and isocitrate, exhibited oscillatory dynamics. This was also found in the model simulations. It is interesting to note that three pairs of conjugate-complex eigenvalues were observed for the Jacobian matrix, which suggests that the system is capable of damped oscillations. The model simulations showed a non-oscillating decrease of fumarate and malate. There was some discrepancy between the simulated time courses for fumarate and malate and the experimentally observed concentrations after 30 min. However, the corresponding standard deviations were large.

The time courses of the experimentally determined cofactors NAD, ATP, and NADP only deviated slightly from their initial values. This means that despite the substantial changes in the metabolite levels in the central carbon metabolism, the homeostatic regulatory machinery of the hepatoma cells only allowed for small changes among the highly linked cofactors: ATP decreased slightly but remained at above 80% of its reference concentration, the NAD level increased only marginally, NADP increased a little more, reaching 143% of its initial value. In contrast to these observations, distinct cofactor dynamics have been observed in similar stimulus response experiments in prokaryotes and yeast [13,21,26].

The experimentally determined reference intermediate levels are provided as supplementary data (cf. Additional file 1).

### Glycolysis Control

Metabolic control patterns are only valid for the physiological condition and cell type used in a particular experiment. For example, Soboll et al. reported significant differences in the control patterns between metabolically inactive and active states in isolated perfused rat liver [34]. In the present study, the reference state of the HepG2 cells was characterized by a sufficient supply of substrates, including glucose [6,7]. Therefore, the hepatoma cells underwent glycolysis rather than gluconeogenesis.

**Figure 3:**
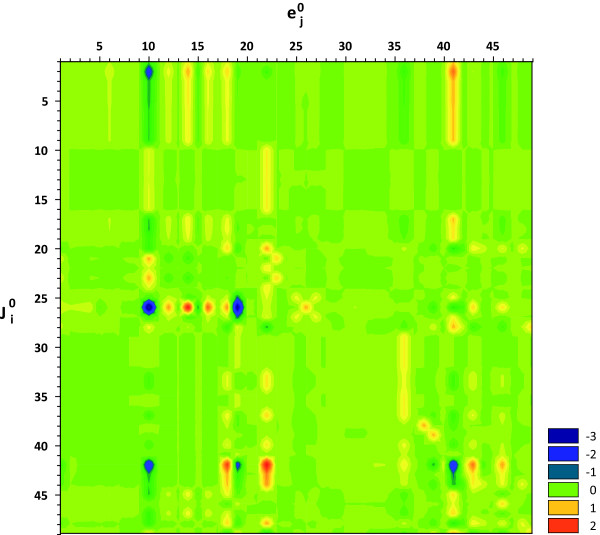
**Flux control coefficients**. The colors in row i and column j indicate the control that enzyme j exerts over the steady flux i. Warm and cold colors denote positive and negative control, respectively. The indices i and j correspond to the reaction numbers shown in Table 1 and Figure 1. The glucose-6-phosphate dehydrogenase (r10) was found to have significant negative control over all glycolytic fluxes, whereas oxidative phosphorylation (r41) exerted positive control (Warburg effect). Furthermore, it is interesting to note that only a few fluxes were found to be significantly stimulated by an increase in the corresponding enzyme level.

**Figure 4:**
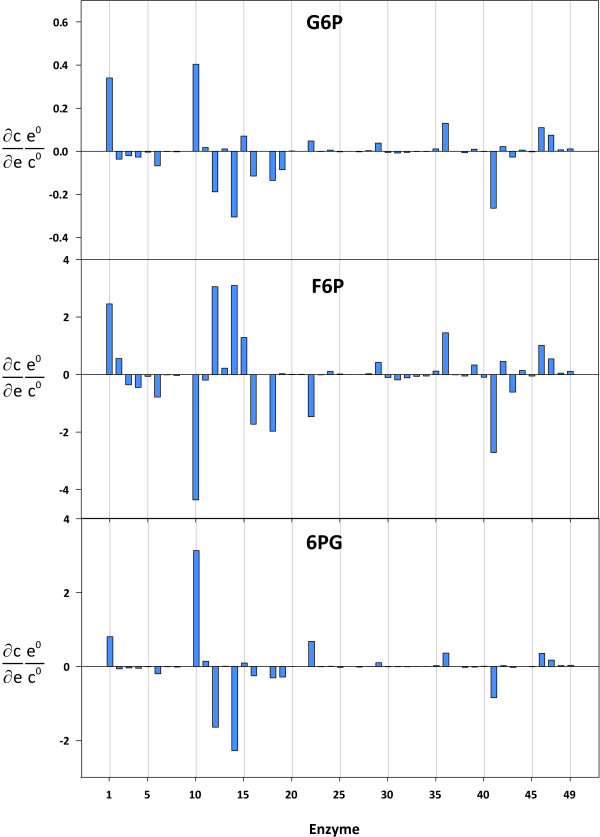
**Concentration control coefficients for glucose-6-phosphate (G6P), fructose-6-phosphate (F6P), and 6-phosphogluconate (6PG)**. The reaction indices correspond to the reaction numbers shown in Table 1 and Figure 1.

The matrix of flux control coefficients is shown in Figure 3 and is also included in the supplementary data section (cf. Additional file 2). The glucose-6-phosphate dehydrogenase enzyme (r10) exerted a substantial negative control over the glycolytic enzymes (r1-r9, r17). The ribose-5-phosphate isomerase (r13) and one transketolase (r15: ribose 5-phosphate + xylulose 5-phosphate = glyceraldehyde 3-phosphate + sedoheptulose 7-phosphate) reaction also had a negative control over the glycolytic flux. In contrast, the phosphogluconate dehydrogenase (r12), the ribulose-phosphate 3-epimerase (r14), and the second transketolase reaction (r16: xylulose 5-phosphate + erythrose 4-phosphate = glyceraldehyde 3-phosphate + fructose-6-phosphate) had a positive control over glycolysis. In each case, the effect on the glucose-6-phosphate isomerase (r2) was far greater than on any other glycolytic enzyme. The flux through this enzyme depends on the concentration of substrate (glucose-6-phosphate), product (fructose-6-phosphate), and inhibitor (6-phosphogluconate). In other words, in order to increase the flux through this enzyme, a perturbation must either lead to an increase in the substrate concentration, or to a decrease in its product and/or inhibitor levels. The corresponding concentration control coefficients were determined in order to find out the effect that was the most significant (cf. Figure 4; cf. Additional file 3). It is interesting to note that the glucose-6-phosphate dehydrogenase (r10) exerted positive and negative control over the glucose-6-phosphate and fructose-6-phosphate levels. However, the enzyme also had positive control over 6-phosphogluconate. Concentration control coefficients provide a quantitative measure of the effects glucose-6-phosphate dehydrogenase had on the relevant substrate, product, and inhibitor levels. Partial flux control coefficients combine this information with the corresponding elasticity value to quantify the fractions to which individual changes in the concentrations of intermediates contribute to the total flux control coefficient.

Table 2 shows the partial flux control coefficients over the glucose-6-phosphate isomerase (r2). The partial flux control coefficients confirmed that 6-phosphogluconate was the key mediator of the negative control exerted by glucose-6-phosphate dehydrogenase. Strong inhibitory effects of 6-phosphogluconate on the glucose-6-phosphate isomerase rate have been reported for various tissues and organisms [42-44]. The phosphogluconate dehydrogenase (r12) and the ribulose-phosphate 3-epimerase (r14) enzymes exerted a negative control over glucose-6-phosphate and 6-phosphogluconate as well as a positive control over fructose-6-phosphate (cf. Figure 4). For both enzymes, the partial flux control coefficients over the flux through the glucose-6-phosphate isomerase (r2), corresponding to the inhibitory 6-phosphogluconate, were found to outweigh the substrate and product effects. In contrast, the second transketolase reaction (r16: xylulose 5-phosphate + erythrose 4-phosphate = glyceraldehyde 3-phosphate + fructose-6-phosphate), which also had positive control over the glucose-6-phosphate isomerase flux, exerted a negative control over all relevant intermediate levels (cf. Figure 4). In this case, though, the impact of 6-phosphogluconate on the flux control was found to play only a minor role. Previous top-down approaches used to quantify the distribution of metabolic control in hepatocytes did not take into account the influences of the pentose-phosphate pathway on the glycolytic flux [8,32,34,45]. Boren et al. recognized the glucose-6-phosphate dehydrogenase (r10) as an interesting target in tumor therapy [46]. They found a flux control coefficient of 0.41 on tumor growth for the glucose-6-phosphate dehydrogenase in mice bearing Ehrlich ascites tumor cells. In addition, cancer cells have a large number of mitochondrial DNA mutations, which possibly results in a dysfunction of the mitochondrial respiratory chain [47]. Carew et al. found a correlation between mitochondrial mutations and an increased generation of reactive oxygen species (ROS) in human leukemia cells [48]. These findings suggest that tumor cells have an elevated demand for reduced NADPH due to the increased scavenging of ROS via glutathione [16]. From the results of the present study, it can further be concluded that the negative control coefficients of glucose-6-phosphate dehydrogenase over the glycolytic fluxes indicate that hepatoma growth is more limited by NADPH than by ATP supply. Moreover, the steady state split ratio between glycolysis and the pentose-phosphate pathway of 57% to 43% [7] provides further evidence for the cells' requirements for reduction equivalents.

**Table 2:** Partial flux control coefficients for glucose-6-phosphate isomerase (r2)

	**Partial flux control coefficient through**			
		
**Enzyme**	**G6P_in_**	**F6P_in_**	**6PG_in_**	**Flux control coefficient**
				
r1	5.64	-2.31	-3.18	0.15
r2	-0.61	-0.52	0.24	0.11
r3	-0.34	0.33	0.14	0.13
r4	-0.44	0.42	0.18	0.16
r5	-0.06	0.06	0.02	0.02
r6	-1.11	0.74	0.76	0.38
r7	-0.02	0.01	0.01	0.01
r8	-0.06	0.03	0.04	0.02
r9	0.00	0.00	0.00	0.00
r10	6.68	4.09	-12.40	-1.63
r11	0.30	0.19	-0.56	-0.07
r12	-3.12	-2.86	6.49	0.50
r13	0.19	-0.21	-0.05	-0.07
r14	-5.06	-2.90	8.98	1.02
r15	1.16	-1.20	-0.40	-0.44
r16	-1.91	1.62	0.97	0.69
r17	0.00	0.00	0.00	0.00
r18	-2.24	1.85	1.20	0.81
r19	-1.42	-0.03	1.12	-0.33
r20	0.02	-0.01	-0.02	-0.01
r21	0.00	0.00	0.00	0.00
r22	0.78	1.37	-2.69	-0.54
r23	-0.02	0.01	0.01	0.00
r24	0.10	-0.10	-0.03	-0.04
r25	-0.04	-0.01	0.06	0.01
r26	-0.01	0.00	0.00	0.00
r27	-0.05	0.00	0.06	0.01
r28	0.04	-0.02	-0.03	-0.01
r29	0.62	-0.39	-0.42	-0.20
r30	-0.08	0.09	0.02	0.03
r31	-0.14	0.17	0.03	0.06
r32	-0.08	0.10	0.02	0.04
r33	-0.02	0.06	-0.02	0.02
r34	-0.02	0.05	-0.02	0.02
r35	0.18	-0.11	-0.12	-0.06
r36	2.15	-1.36	-1.46	-0.67
r37	-0.01	0.01	0.00	0.00
r38	-0.10	0.05	0.08	0.03
r39	0.17	-0.32	0.05	-0.09
r40	-0.03	0.09	-0.03	0.03
r41	-4.38	2.55	3.32	1.48
r42	0.37	-0.42	-0.10	-0.15
r43	-0.45	0.57	0.09	0.21
r44	0.09	-0.13	0.00	-0.04
r45	-0.05	0.04	0.03	0.02
r46	1.82	-0.94	-1.40	-0.53
r47	1.24	-0.51	-0.70	0.03
r48	0.12	-0.05	-0.11	-0.03
r49	0.18	-0.10	-0.14	-0.06

Partial flux control coefficients are only shown for metabolites that have an effect on the glucose-6-phosphate isomerase (r2), i.e. that have non-zero elasticities. The sum of the partial flux control coefficients over all intermediates equals the flux control coefficient. With regard to the control of an enzyme over its own flux, the flux control coefficient equals the sum of the partial flux control coefficients plus one.

In accordance with the negative control over the glycolytic fluxes, glucose-6-phosphate dehydrogenase (r10) was found to have substantial negative control over the formation of glycerol (r26).

Lactate dehydrogenase (r18) had a substantial positive control over the glycolytic fluxes. Ainscow and Brand reported positive control of lactate production on glycolysis in primary hepatocytes isolated from fed rats [8]. The control coefficient (0.12) was smaller than the values of the individual glycolysis enzymes determined in this study (0.43 - 0.8). However, hepatoma cells, like most tumor cells, produce large amounts of lactate under aerobic conditions. Consequently, the lactate dehydrogenase enzyme is likely to be closer to saturation in tumor cells than in primary cells. An enzyme with a low elasticity coefficient, i.e. an enzyme operating close to saturation, hardly responds to changes in the levels of its substrate and/or product molecules. Therefore, saturated enzymes exhibit a larger flux control compared to unsaturated enzymes [49].

The pyruvate dehydrogenase complex (r36) and the pyruvate secretion step (r38) had a negative control over the glycolytic flux. For pyruvate oxidation, Ainscow and Brand also observed a negative control over glycolysis in rat hepatocytes [8]. Increasing the flux through the pyruvate dehydrogenase complex led to an increased flux through the TCA cycle, which, in turn, increased the intracellular NADH/NAD ratio. Besides, the pyruvate dehydrogenase complex exerted negative control over the lactate dehydrogenase enzyme (-0.35), which leads to an even higher NADH/NAD ratio. Pyruvate secretion had negative control over both the lactate dehydrogenase (-0.29) and the pyruvate dehydrogenase complex (-0.03). Consequently, the negative control over glycolysis exerted by the pyruvate dehydrogenase complex was found to be more substantial than the negative control of the pyruvate transportation step.

Ainscow and Brand observed a negative control for oxidative phosphorylation over the glycolytic flux in primary rat hepatocytes (flux control coefficient of -0.26) [8]. This was attributed to the Pasteur effect, where the increased activity of oxidative phosphorylation slows down glycolysis. In an analysis of the partial flux control coefficients of the glycolysis block, Ainscow and Brand found for isolated rat hepatocytes that the Pasteur effect was mostly due to an increase in ATP, which was opposed by a decreasing NADH/NAD ratio [32]. However, in the case of HepG2 cells, oxidative phosphorylation (r41) had a substantial positive control of glycolysis, with control coefficients ranging from 0.82 to 1.48. This suggests that the respiration rate has a limiting effect on the growth of hepatomas. This has also been proposed for prokaryotic systems [50,51]. Furthermore, Lo et al. reported only low respiration for rapidly growing, poorly differentiated hepatic tumors, an effect which was ascribed to the loss of mitochondria during dedifferentiation [52]. The aforementioned increased mitochondrial DNA mutation rate can also lead to a dysfunction of the mitochondrial respiratory chain [47]. Warburg was the first to describe what is today known as the Warburg effect or aerobic glycolysis: In contrast to normal liver tissue, liver cancer cells have an increased glycolytic flux in the presence of oxygen [53]. The Warburg effect is often observed in tumor tissue. In fact, the elevated glycolytic flux of malignant cells is increasingly recognized as a promising target for the treatment of cancer [54,55].

### Control of the Pentose Phosphate Pathway

The control of the pentose-phosphate pathway (r10-r16, r24) depends to a great extent on the demand for reduction equivalents (r22) and the glucose-6-phosphate dehydrogenase enzyme (r10). This means that flux control coefficients of 0.63 and 0.41 of the individual fluxes in the pentose-phosphate shunt were observed for NADPH consumption (r22) and the glucose-6-phosphate dehydrogenase enzyme (r10), respectively. Kather et al. also reported that the glucose-6-phosphate dehydrogenase and the NADPH/NADP ratio control the pentose-phosphate pathway of isolated fat-cells [56]. Sabate et al. described a kinetic pentose-phosphate pathway model for fasted rat livers [37], which, however, does not take into consideration NADPH consumption. The authors concluded that the pentose-phosphate pathway fluxes were mainly regulated by the glucose-6-phosphate dehydrogenase and transketolase reactions.

The predominant control of the pentose-phosphate pathway by the glucose-6-phosphate dehydrogenase (r10) and the demand for reduction equivalents (r22) is also interesting with respect to the discussion of modular structures in metabolic networks. Using dynamic modeling and experimental observations of *in vivo *metabolite dynamics, Vaseghi et al. concluded that in *Saccharomyces cerevisiae *the pentose-phosphate pathway acts as a functional unit that is controlled by the demand for biosynthesis and is modulated by the energy state of the cell [57]. In accordance with the yeast enzyme, the flux through the human glucose-6-phosphate dehydrogenase is also modulated by the cellular ATP level [58]. In this work, the elasticity value of the glucose-6-phosphate dehydrogenase with respect to ATP was determined to -0.7. Together with the observed control principles, this suggests a similar dynamic regulation scheme in hepatoma cells.

### Control of the Tricarboxylic Acid Cycle

**Figure 5:**
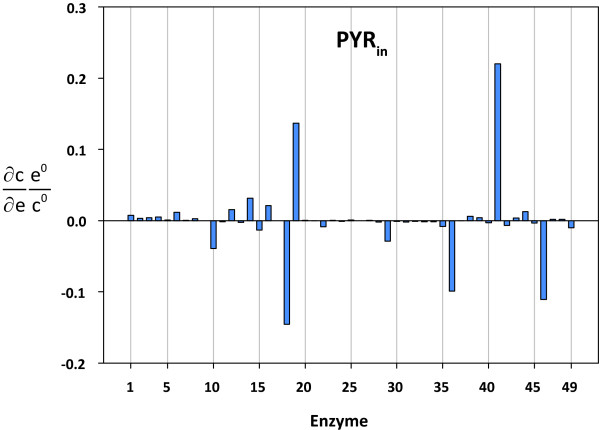
**Concentration control coefficients for intracellular pyruvate**. The reaction indices correspond to the reaction numbers shown in Table 1 and Figure 1.

Ainscow and Brand applied top-down methods to elucidate metabolic control patterns in isolated rat hepatocytes. They included only one common reaction block for pyruvate transport, TCA cycle, and five-sixths of the respiratory chain [8,32]. Thus, the approach used in the present study allows a more detailed investigation of the control patterns of the TCA cycle. The glucose-6-phosphate dehydrogenase (r10) exerted little positive and substantial negative control over the first (r29-r32) and last (r33, r34, r40, r43) fluxes in the TCA cycle, respectively. Partial flux control coefficients were calculated in order to find out where these different effects stem from. The results are listed in Table 3. As can be seen, the negative control over the malic enzyme (r43) was due to an increased NADPH level (-1.13) and a decreased NADP (-0.63) level. These effects were partially compensated by lower intracellular pyruvate (0.32) and increased malate (0.45) levels. The increase in the malate concentration, in turn, was the key mediator of the negative control of the glucose-6-phosphate dehydrogenase (r10) over the flux through the fumarate hydratase (r34). A decreased flux through this enzyme was accompanied by elevated fumarate levels, which resulted in a substantial negative flux control coefficient for the succinate dehydrogenase (r40). Likewise, the flux through the succinate-CoA ligase enzyme (r33) was negatively controlled by its product succinate. To summarize, the positive and negative control over the NADPH and NADP levels of the glucose-6-phosphate dehydrogenase (r10) enzyme led to a negative control of the flux through the malic enzyme (r43). The negative control of the adjacent TCA cycle fluxes was mediated by elevated product levels. Consequently, the consumption of reduction equivalents (r22) was expected to lead to a complementary control pattern, and this was indeed the case. Similarly, the lactate dehydrogenase enzyme (r18) exerted a positive control over the final fluxes of the TCA cycle. In contrast to the glucose-6-phosphate dehydrogenase (r10) and the consumption of reduction equivalents (r22), the lactate dehydrogenase enzyme had a minor effect on the cellular NADPH/NADP ratio. However, as expected, lactate dehydrogenase exerted a substantial negative control over the intracellular pyruvate concentration. Figure 5 depicts the concentration control coefficients for intracellular pyruvate. The negative control of lactate dehydrogenase (r22) over the intracellular pyruvate level results in a strong positive control over the malic enzyme flux (1.17; cf. Table 3). The positive control was mediated through the downstream reactions in the TCA cycle by decreasing the product levels. The ATP-consuming reaction (r19) had a positive control over the first fluxes in the TCA cycle (citrate synthase, r29; aconitate hydratase, r30, r31; isocitrate dehydrogenase, r32) as well as over the flux through the pyruvate dehydrogenase (r36). The flux control coefficient for the pyruvate dehydrogenase flux of 0.29 (cf. Additional file 2) was mainly due to increased NAD (0.2) and pyruvate (0.14) levels and, to a lesser extent, a lower NADH level (0.08). These effects were opposed by an increased acetyl-CoA level (-0.13). The elevated intracellular pyruvate level mediated a negative control over the malic enzyme (-1.1). As for the pyruvate dehydrogenase enzyme, the negative control of the malic enzyme was accompanied by an increased malate level, which led to a negative control of the flux through the fumarate hydratase enzyme. A substantial positive control of the complete TCA cycle was observed for the pyruvate dehydrogenase complex (r36). Using a top-down approach, Ainscow and Brand observed in rat hepatocytes that the pyruvate oxidation block had a positive control over itself [8]. The oxidative phosphorylation reaction (r41) had the highest positive control over the intracellular steady state pyruvate level (cf. Figure 5).

**Table 3:** Partial flux control coefficients for succinate-CoA ligase (r33), fumarate hydratase (r34), succinate dehydrogenase (r40), and the malic enzyme (r43)

	r33					r34			r40					r43				
	
	Partial flux control coefficient through				Flux control coefficient	Partial flux control coefficient through		Flux control coefficient	Partial flux control coefficient through				Flux control coefficient	Partial flux control coefficient through				Flux control coefficient
								
Enzyme	ATP_in_	SUC_in_	SUCCOA_in_	ADP_in_		MAL_in_	FUM_in_		NAD_in_	SUC_in_	FUM_in_	NADH_in_		PYR_in_	NADP_in_	MAL_in_	NADPH_in_	
r1	-0.005	-0.196	0.018	-0.002	-0.185	-0.598	0.414	-0.185	0.000	0.126	-0.247	-0.063	-0.185	-0.060	-0.127	0.085	-0.227	-0.328
r2	-0.003	0.003	-0.001	-0.001	-0.002	0.089	-0.091	-0.002	0.000	-0.002	0.055	-0.055	-0.002	-0.025	0.017	-0.013	0.030	0.009
r3	-0.003	-0.009	0.000	-0.002	-0.014	0.061	-0.075	-0.014	0.000	0.006	0.045	-0.064	-0.014	-0.032	0.011	-0.009	0.019	-0.010
r4	-0.004	-0.012	0.000	-0.002	-0.018	0.077	-0.094	-0.018	0.000	0.008	0.056	-0.081	-0.018	-0.040	0.014	-0.011	0.024	-0.013
r5	-0.001	-0.002	0.000	0.000	-0.002	0.011	-0.013	-0.002	0.000	0.001	0.008	-0.011	-0.002	-0.005	0.002	-0.002	0.003	-0.002
r6	-0.009	-0.028	0.000	-0.005	-0.042	0.178	-0.220	-0.042	-0.001	0.018	0.132	-0.191	-0.042	-0.093	0.031	-0.025	0.056	-0.032
r7	0.000	0.000	0.000	0.000	-0.001	0.003	-0.003	-0.001	0.000	0.000	0.002	-0.003	-0.001	-0.001	0.000	0.000	0.001	0.000
r8	0.000	-0.006	0.001	0.000	-0.006	-0.026	0.020	-0.006	0.000	0.004	-0.012	0.002	-0.006	-0.021	0.002	0.004	0.003	-0.013
r9	0.000	0.000	0.000	0.000	0.000	0.000	0.000	0.000	0.000	0.000	0.000	0.000	0.000	0.000	0.000	0.000	0.000	0.000
r10	0.036	-0.544	0.064	0.019	-0.425	-3.145	2.720	-0.425	0.003	0.349	-1.624	0.847	-0.425	0.315	-0.631	0.449	-1.131	-0.998
r11	0.002	-0.025	0.003	0.001	-0.019	-0.143	0.123	-0.019	0.000	0.016	-0.074	0.038	-0.019	0.014	-0.029	0.020	-0.051	-0.045
r12	-0.012	-0.047	0.001	-0.006	-0.065	0.196	-0.261	-0.065	-0.001	0.030	0.156	-0.250	-0.065	-0.125	0.033	-0.028	0.060	-0.060
r13	0.002	0.006	0.000	0.001	0.009	-0.034	0.043	0.009	0.000	-0.004	-0.026	0.038	0.009	0.019	-0.006	0.005	-0.011	0.006
r14	-0.026	-0.090	0.002	-0.013	-0.127	0.422	-0.549	-0.127	-0.002	0.058	0.328	-0.510	-0.127	-0.253	0.073	-0.060	0.131	-0.108
r15	0.011	0.032	0.000	0.006	0.048	-0.209	0.257	0.048	0.001	-0.020	-0.153	0.221	0.048	0.109	-0.037	0.030	-0.066	0.035
r16	-0.017	-0.050	0.000	-0.009	-0.075	0.322	-0.398	-0.075	-0.001	0.032	0.238	-0.344	-0.075	-0.170	0.057	-0.046	0.102	-0.056
r17	0.000	0.000	0.000	0.000	0.000	-0.001	0.001	0.000	0.000	0.000	0.000	0.000	0.000	-0.001	0.000	0.000	0.000	0.000
r18	-0.019	0.532	-0.058	-0.010	0.446	2.173	-1.728	0.446	-0.001	-0.342	1.031	-0.243	0.446	1.168	0.052	-0.310	0.093	1.003
r19	0.061	-0.324	0.048	0.032	-0.182	-2.522	2.340	-0.182	0.004	0.208	-1.397	1.004	-0.182	-1.099	0.004	0.360	0.007	-0.729
r20	0.000	0.000	-0.001	0.000	-0.001	0.004	-0.005	-0.001	0.000	0.000	0.003	-0.004	-0.001	-0.002	0.000	-0.001	-0.001	-0.004
r21	0.000	0.000	0.000	0.000	0.000	-0.001	0.001	0.000	0.000	0.000	-0.001	0.001	0.000	0.000	0.000	0.000	0.000	0.000
r22	0.002	0.751	-0.074	0.001	0.680	2.787	-2.107	0.680	0.000	-0.482	1.258	-0.096	0.680	0.069	0.579	-0.398	1.037	1.287
r23	0.000	0.000	0.000	0.000	0.000	0.000	0.000	0.000	0.000	0.000	0.000	0.000	0.000	-0.001	0.000	0.000	0.001	0.000
r24	0.001	0.003	0.000	0.000	0.004	-0.018	0.022	0.004	0.000	-0.002	-0.013	0.019	0.004	0.009	-0.003	0.003	-0.006	0.003
r25	0.000	-0.002	0.000	0.000	-0.002	-0.012	0.010	-0.002	0.000	0.001	-0.006	0.003	-0.002	-0.007	0.001	0.002	0.001	-0.004
r26	0.000	0.000	0.000	0.000	0.000	-0.002	0.002	0.000	0.000	0.000	-0.001	0.001	0.000	-0.001	0.000	0.000	0.000	0.000
r27	0.000	0.000	0.000	0.000	0.000	0.007	-0.006	0.000	0.000	0.000	0.004	-0.003	0.000	-0.002	0.001	-0.001	0.002	0.000
r28	0.000	0.005	-0.001	0.000	0.005	0.023	-0.019	0.005	0.000	-0.003	0.011	-0.003	0.005	0.018	-0.001	-0.003	-0.002	0.011
r29	0.004	0.146	-0.008	0.002	0.144	0.584	-0.440	0.144	0.000	-0.094	0.263	-0.024	0.144	0.230	-0.018	-0.083	-0.032	0.098
r30	-0.001	0.010	-0.001	0.000	0.008	0.077	-0.069	0.008	0.000	-0.006	0.041	-0.027	0.008	0.009	0.003	-0.011	0.005	0.005
r31	-0.002	0.018	-0.001	-0.001	0.014	0.141	-0.127	0.014	0.000	-0.011	0.076	-0.050	0.014	0.016	0.005	-0.020	0.009	0.009
r32	-0.001	0.010	-0.001	-0.001	0.008	0.084	-0.075	0.008	0.000	-0.007	0.045	-0.030	0.008	0.009	0.003	-0.012	0.005	0.005
r33	-0.001	-0.924	-0.007	0.000	0.068	-0.755	0.823	0.068	0.000	0.592	-0.492	-0.033	0.068	0.014	0.000	0.108	-0.001	0.121
r34	-0.001	0.071	-0.007	0.000	0.063	-0.703	-0.234	0.063	0.000	-0.046	0.139	-0.030	0.063	0.013	0.000	0.100	-0.001	0.112
r35	0.001	0.042	-0.002	0.001	0.041	0.168	-0.126	0.041	0.000	-0.027	0.075	-0.007	0.041	0.066	-0.005	-0.024	-0.009	0.028
r36	0.013	0.503	-0.026	0.007	0.497	2.012	-1.515	0.497	0.000	-0.323	0.904	-0.084	0.497	0.793	-0.061	-0.287	-0.109	0.337
r37	0.000	-0.013	0.015	0.000	0.002	-0.002	0.004	0.002	0.000	0.008	-0.002	-0.004	0.002	0.001	0.000	0.000	0.000	0.002
r38	-0.001	-0.014	0.002	0.000	-0.013	-0.070	0.057	-0.013	0.000	0.009	-0.034	0.012	-0.013	-0.049	0.003	0.010	0.005	-0.031
r39	0.003	-0.061	0.030	0.002	-0.027	-0.360	0.334	-0.027	0.000	0.039	-0.199	0.133	-0.027	-0.034	-0.007	0.051	-0.013	-0.003
r40	-0.001	0.119	-0.011	-0.001	0.106	-1.177	1.284	0.106	0.000	-0.076	-0.766	-0.051	0.106	0.022	-0.001	0.168	-0.001	0.188
r41	-0.040	-0.493	0.062	-0.021	-0.492	-2.273	1.782	-0.492	0.001	0.316	-1.064	0.255	-0.492	-1.765	0.133	0.324	0.239	-1.069
r42	0.004	-0.032	0.022	0.002	-0.004	-0.235	0.231	-0.004	0.000	0.021	-0.138	0.112	-0.004	0.053	-0.013	0.034	-0.023	0.050
r43	-0.006	0.421	-0.043	-0.003	0.369	1.774	-1.405	0.369	-0.001	-0.270	0.839	-0.199	0.369	-0.030	0.006	-0.253	0.011	0.735
r44	0.001	-0.051	0.011	0.001	-0.038	-0.239	0.201	-0.038	0.000	0.032	-0.120	0.049	-0.038	-0.100	-0.002	0.034	-0.004	-0.073
r45	0.000	0.013	-0.001	0.000	0.011	0.053	-0.042	0.011	0.000	-0.008	0.025	-0.006	0.011	0.028	0.001	-0.007	0.002	0.024
r46	0.009	0.260	-0.033	0.005	0.241	1.285	-1.044	0.241	-0.001	-0.167	0.623	-0.214	0.241	0.888	-0.052	-0.183	-0.094	0.558
r47	-0.001	-0.043	0.004	-0.001	-0.041	-0.132	0.091	-0.041	0.000	0.028	-0.055	-0.014	-0.041	-0.013	-0.028	0.019	-0.050	-0.072
r48	0.001	-0.002	-0.005	0.000	-0.005	0.022	-0.027	-0.005	0.000	0.001	0.016	-0.023	-0.005	-0.013	-0.003	-0.003	-0.005	-0.024
r49	0.001	0.022	-0.003	0.001	0.022	0.107	-0.086	0.022	0.000	-0.014	0.051	-0.015	0.022	0.080	-0.005	-0.015	-0.010	0.050

Partial flux control coefficients are only shown for metabolites with non-zero elasticities. The sum of the partial flux control coefficients over all intermediates equals the flux control coefficient. For the control of an enzyme over its own flux, the flux control coefficient equals the sum of the partial flux control coefficients plus one.

Reaction r42 describes the exchange of alpha-ketoglutarate with the biomass. In the dynamic network model, the rate of r42 depends only on the level of its product alpha-ketoglutarate. The corresponding elasticity coefficient was determined to -0.5. This means that changes in the level of alpha-ketoglutarate are directly mirrored in changes in the flux through the r42 reaction, and, thus, r42 was found to be strongly influenced by several enzymes.

### Control of Lactate Dehydrogenase, NADPH Consumption, and Oxidative Phosphorylation

**Figure 6:**
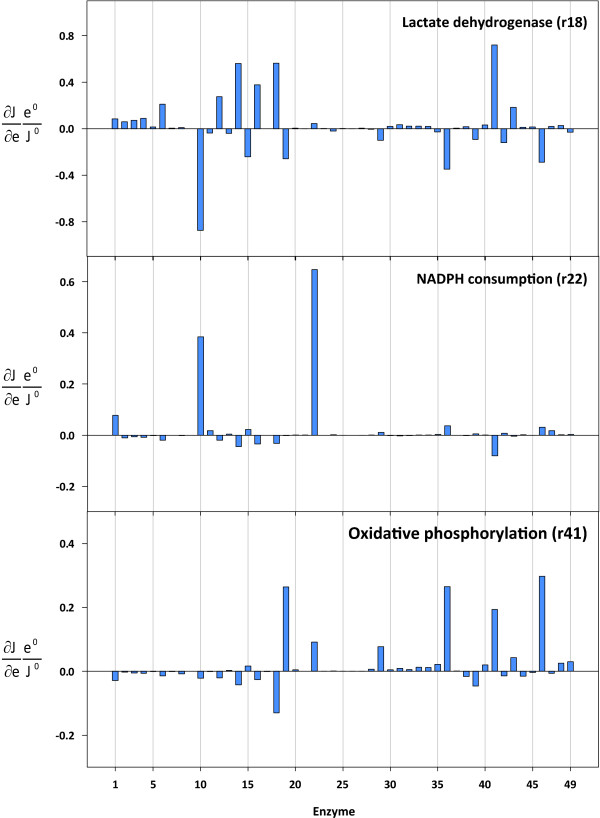
**Flux control coefficients for lactate dehydrogenase (r18), NADPH consumption (r22), and oxidative phosphorylation (r41)**. The reaction indices correspond to the reaction numbers shown in Table 1 and Figure 1.

Figure 6 depicts the flux control coefficients for lactate dehydrogenase (r18), NADPH consumption (r22), and oxidative phosphorylation (r41). The glycolytic enzymes had positive control over lactate production. However, the effect was less significant compared to primary hepatocytes that were isolated from fed rats [8]. The pentose-phosphate pathway exerted a significant control over the glycolytic flux and thus had substantial control over the flux through the lactate dehydrogenase enzyme (r18). The corresponding partial flux control coefficients are listed in Table 4. Most of the control of the pentose phosphate pathway was mediated by its influence on the NADH level. Changes in NAD and pyruvate concentrations also contributed to the total flux control coefficient, albeit to a lesser extent. Similarly to the control pattern observed in rat hepatocytes [8], the pyruvate dehydrogenase complex (r36) exerted a negative control over the lactate dehydrogenase flux. The same authors also emphasized the importance of the pyruvate level with regard to the lactate production rate [32]. Ainscow and Brand found that lactate dehydrogenase (r18) had little control over its own flux, as increased activity was strongly counteracted by low pyruvate levels. The effect exerted by decreasing pyruvate levels could also be observed in hepatoma cells, but was less pronounced. Therefore, the lactate dehydrogenase had more control over its own flux. Furthermore, in contrast to the situation observed in primary hepatocytes, the oxidative phosphorylation (r41) in hepatoma cells had substantial positive control over the lactate production rate. This was mainly due to its increasing effect on the pyruvate level (0.95).

**Table 4:** Partial flux control coefficients for lactate dehydrogenase (r18) and oxidative phosphorylation (r41)

	r18					r41				
	
	Partial flux control coefficient through					Partial flux control coefficient through				
				
Enzyme	NAD_in_	PYR_in_	LAC_in_	NADH_in_	Flux control coefficient	ATP_in_	NAD_in_	ADP_in_	NADH_in_	Flux control coefficient
r1	0.011	0.032	-0.002	0.043	0.084	-0.012	0.001	-0.060	0.042	-0.028
r2	0.009	0.014	-0.001	0.038	0.059	-0.007	0.001	-0.034	0.037	-0.003
r3	0.011	0.017	-0.002	0.044	0.071	-0.008	0.002	-0.041	0.044	-0.005
r4	0.014	0.022	-0.002	0.056	0.089	-0.011	0.002	-0.052	0.055	-0.006
r5	0.002	0.003	0.000	0.008	0.012	-0.001	0.000	-0.007	0.008	-0.001
r6	0.032	0.050	-0.005	0.132	0.209	-0.025	0.005	-0.124	0.129	-0.015
r7	0.000	0.001	0.000	0.002	0.003	0.000	0.000	-0.002	0.002	0.000
r8	0.000	0.012	0.000	-0.002	0.009	-0.001	0.000	-0.005	-0.002	-0.008
r9	0.000	0.000	0.000	0.000	0.000	0.000	0.000	0.000	0.000	0.000
r10	-0.142	-0.170	0.021	-0.584	-0.875	0.096	-0.020	0.476	-0.573	-0.021
r11	-0.006	-0.008	0.001	-0.027	-0.040	0.004	-0.001	0.022	-0.026	-0.001
r12	0.042	0.067	-0.007	0.173	0.275	-0.033	0.006	-0.163	0.169	-0.020
r13	-0.006	-0.010	0.001	-0.026	-0.041	0.005	-0.001	0.024	-0.026	0.003
r14	0.086	0.137	-0.014	0.352	0.561	-0.068	0.012	-0.331	0.345	-0.042
r15	-0.037	-0.059	0.006	-0.152	-0.242	0.029	-0.005	0.142	-0.149	0.017
r16	0.058	0.092	-0.009	0.237	0.377	-0.045	0.008	-0.222	0.233	-0.026
r17	0.000	0.000	0.000	0.000	0.000	0.000	0.000	0.000	0.000	0.000
r18	0.041	-0.632	-0.014	0.168	0.564	-0.050	0.006	-0.250	0.165	-0.130
r19	-0.168	0.594	0.006	-0.693	-0.261	0.162	-0.024	0.805	-0.679	0.264
r20	0.001	0.001	0.000	0.003	0.005	0.000	0.000	0.001	0.003	0.005
r21	0.000	0.000	0.000	-0.001	-0.001	0.000	0.000	0.001	-0.001	0.000
r22	0.016	-0.037	-0.001	0.066	0.044	0.005	0.002	0.020	0.065	0.091
r23	0.000	0.001	0.000	0.000	0.001	0.000	0.000	0.000	0.000	0.000
r24	-0.003	-0.005	0.000	-0.013	-0.020	0.002	0.000	0.012	-0.013	0.001
r25	0.000	0.004	0.000	-0.002	0.002	0.000	0.000	0.002	-0.002	0.000
r26	0.000	0.000	0.000	-0.001	-0.001	0.000	0.000	0.001	-0.001	0.000
r27	0.001	0.001	0.000	0.002	0.004	0.000	0.000	-0.002	0.002	0.001
r28	0.001	-0.009	0.000	0.002	-0.006	0.001	0.000	0.004	0.002	0.007
r29	0.004	-0.125	0.002	0.017	-0.101	0.010	0.001	0.050	0.017	0.077
r30	0.005	-0.005	0.000	0.019	0.018	-0.002	0.001	-0.012	0.019	0.005
r31	0.008	-0.008	-0.001	0.035	0.034	-0.004	0.001	-0.022	0.034	0.009
r32	0.005	-0.005	0.000	0.021	0.020	-0.003	0.001	-0.013	0.020	0.006
r33	0.005	-0.008	0.000	0.022	0.020	-0.002	0.001	-0.008	0.022	0.013
r34	0.005	-0.007	0.000	0.021	0.018	-0.002	0.001	-0.008	0.020	0.012
r35	0.001	-0.036	0.001	0.005	-0.029	0.003	0.000	0.014	0.005	0.022
r36	0.014	-0.429	0.008	0.058	-0.348	0.035	0.002	0.171	0.057	0.265
r37	0.001	-0.001	0.000	0.003	0.003	0.000	0.000	-0.001	0.003	0.001
r38	-0.002	0.026	0.000	-0.008	0.016	-0.001	0.000	-0.007	-0.008	-0.016
r39	-0.022	0.018	0.002	-0.092	-0.094	0.008	-0.003	0.039	-0.090	-0.046
r40	0.009	-0.012	-0.001	0.035	0.031	-0.003	0.001	-0.013	0.034	0.020
r41	-0.043	0.954	-0.017	-0.176	0.718	-0.105	-0.006	-0.523	-0.172	0.193
r42	-0.019	-0.028	0.003	-0.078	-0.122	0.011	-0.003	0.054	-0.076	-0.014
r43	0.033	0.016	-0.004	0.137	0.183	-0.016	0.005	-0.081	0.135	0.042
r44	-0.008	0.054	0.000	-0.034	0.012	0.003	-0.001	0.016	-0.033	-0.015
r45	0.001	-0.015	0.024	0.004	0.014	-0.001	0.000	-0.006	0.004	-0.003
r46	0.036	-0.480	0.007	0.148	-0.289	0.025	0.005	0.122	0.145	0.297
r47	0.002	0.007	0.000	0.010	0.019	-0.003	0.000	-0.013	0.009	-0.006
r48	0.004	0.007	-0.001	0.016	0.026	0.002	0.001	0.008	0.015	0.025
r49	0.003	-0.043	0.001	0.010	-0.030	0.003	0.000	0.016	0.010	0.030

Partial flux control coefficients are only shown for metabolites having non-zero elasticities. The sum of the partial flux control coefficients for all intermediates equals the flux control coefficient. With regard to the control of an enzyme over its own flux, the flux control coefficient equals the sum of the partial flux control coefficients plus one.

NADPH consumption (r22) was mainly controlled by itself (0.65). However, it is important to note that it is not only the demand of NADPH alone that affects the NADPH consumption flux, but also the supply of NADPH (0.38). This means that an increase in NADPH production yields an increase in NADPH consumption, i.e. a stimulation of biosynthetic reactions. Put differently, the dependence of NADPH demand on NADPH supply provides further evidence for the hypothesis that tumor growth is limited by NADPH production.

Numerous papers have dealt with the control of oxidative phosphorylation in isolated rat liver cells [8,34,59]. The results found for hepatoma cells agree with those previously found: Inhibition of oxygen consumption resulting from an increased flux through glycolysis (Crabtree effect) was low [8], the consumption of ATP (r19) and the pyruvate dehydrogenase complex (r36) positively controlled oxidative phosphorylation [8,59] and oxidative phosphorylation had a strong control over its own flux [8,34].

### Concentration Control over NADPH, NADH, ATP, and NAD

**Figure 7:**
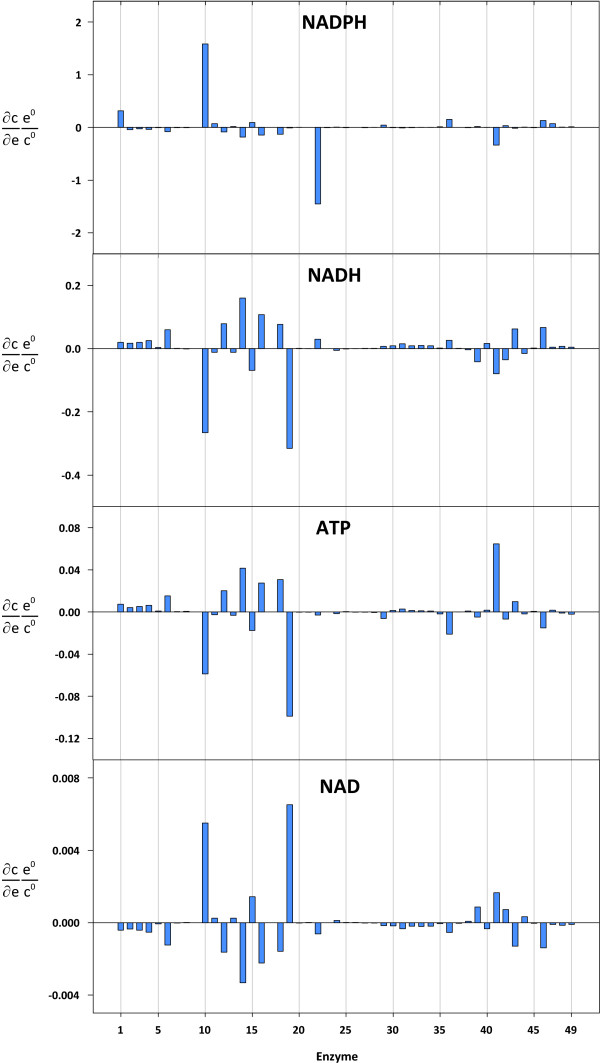
**Concentration control coefficients for NADPH, NADH, ATP, and NAD**. The reaction indices correspond to the reaction numbers shown in Table 1 and Figure 1.

The steady state NADPH level was found to be very sensitive to two reactions - the glucose-6-phosphate dehydrogenase (r10) and the consumption of reduction equivalents (r22) (concentration control coefficients for NADPH, NADH, ATP, and NAD are shown in Figure 7). In both cases, the absolute values were above 1, i.e. 1.58 for r10 and -1.45 for r22. This means that the NADPH level was equally controlled by supply and demand. In addition, NADPH responded moderately to changes in the glucokinase (r1; 0.32) and oxidative phosphorylation (r41; -0.33) reactions. Apart from these, NADPH did not react significantly to changes in the levels of other enzymes.

With respect to glycolysis and the pentose-phosphate pathway, the control distribution for ATP and NADH levels were similar. However, the values were proportionally lower for ATP, which was due to the stoichiometry, i.e. a P/O ratio of 2.5 was assumed for NADH (cf. sub-section model reconstruction). In both cases, the glycolytic enzymes (r1-r9, r17) had a positive, albeit low, control over the cofactors. As expected, the glucose-6-phosphate dehydrogenase enzyme (r10) had a negative control over both intermediates, i.e. control coefficients of -0.06 for ATP and -0.27 for NADH, respectively. In contrast, the r12 (phosphogluconate dehydrogenase), r14 (ribulose-phosphate 3-epimerase), and r16 (transketolase: xylulose 5-phosphate + erythrose 4-phosphate = glyceraldehyde 3-phosphate + fructose-6-phosphate) enzymes in the pentose-phosphate pathway had a positive control over the NADH and ATP levels. A major characteristic of cancer cells, including the hepatoma cells analyzed in this study, is the secretion of lactate under aerobic conditions [53]. The aerobic production of lactate leads to an increase in the amount of ATP produced per time unit at the expense of a poorer yield coefficient. It was therefore assumed, and confirmed, that the lactate dehydrogenase enzyme had a positive control over the ATP level. The situation was different for NADH. On the one hand, increasing the flux through the lactate dehydrogenase enzyme decreases the NADH level by reducing pyruvate to lactate. On the other hand, it allows for an increased glycolytic flux, which leads to an increased level of NADH. Under the physiological conditions investigated in the present study, the latter effect was found to outweigh the former, which is the reason why a positive control coefficient was observed. ATP and NADH responded substantially and negatively to changes in the ATP-consuming reaction (r19; ATP: -0.1 and NADH: -0.31). It is not surprising that the control coefficients for the NAD level were complementary to those for NADH. However, the values were two orders of magnitude lower for NAD. The lower values for NAD compared to NADH are due to the normalization to the reference concentrations, which are lower for NADH.

### Partial Internal Response Coefficients

Partial internal response coefficients quantify system responses to changes in the concentration of intermediates [32,60,61]. An asymptotically stable metabolic network operating at steady state will counteract an increase in one of its metabolites by either increasing the consumption of that metabolite, decreasing the production or by some combination thereof. In this context, partial internal response coefficients allow for the assessment of the relevance of individual reactions in counteracting a perturbation in order to restore the steady state. The internal response coefficients for the system under discussion are listed in Additional file 4. The cellular responses to elevated pyruvate level were in line with previous findings in rat hepatocytes, i.e. elevated pyruvate levels were mainly counteracted by an increased flux through the lactate dehydrogenase enzyme (r18) [32]. However, the value of the coefficient was lower in hepatoma cells, i.e. -0.63 compared to -0.84 as determined for rat hepatocytes. In HepG2 cells, a fraction of the additional pyruvate was secreted into the extracellular space (-0.23), another fraction was consumed by the pyruvate dehydrogenase complex (-0.1). In isolated rat hepatocytes, an increase in glucose-6-phosphate was counteracted by an increased release of glucose (-0.81) and by less glycogen being broken down (-0.17). The coefficient resulting from glycolytic degradation was negligible [32]. In hepatoma cells, the control pattern was fundamentally different: An increase in glycogen synthesis played only a negligible role in counteracting elevated glucose-6-phosphate levels. The elevated glucose-6-phosphate levels were mainly counteracted by a reduced synthesis rate (r1, glucokinase; -0.75) and an increased flux through glucose-6-phosphate isomerase (-0.61). Furthermore, the internal response coefficient for the flux of glucose-6-phosphate through glucose-6-phosphate dehydrogenase was positive (0.36). It is interesting to note that in the cases of 1,3-bisphospho-glycerate, glycerate-3-phosphate, glycerate-2-phosphate, and phosphoenolpyruvate, an increase in intermediate was counteracted almost entirely by increased consumption. Ainscow and Brand attributed the high internal response coefficients for the flux of pyruvate through lactate dehydrogenase in rat hepatocytes to the fact that lactate dehydrogenase was working close to equilibrium [32]. Similarly, in hepatoma cells the reactions in the lower part of glycolysis are possibly also close to equilibrium. Holzhütter et al. reported near equilibrium operation for the lower part of glycolysis in human erythrocytes [60].

## Conclusions

This study dealt with the quantitative assessment of the dynamics and control of the central carbon metabolism in hepatoma cells. Metabolite time-series data analyzed in a stimulus response experiment revealed substantial changes in the concentrations of intermediates, and were used for identifying network dynamics. Control analysis was applied in order to break down the internal control structure of the central carbon metabolism in hepatoma cells. In comparison to previous top-down approaches, this study enabled the more detailed analysis of the underlying control patterns. Rather than describing how sub-systems interact with each other, the control distribution approach used quantifies the influences the individual enzymes have on each other. It was possible to unravel many different interactions: Glucose-6-phosphate dehydrogenase had a substantial negative control over the glycolytic flux. Partial flux control coefficients were determined in order to assess the importance of the individual interactions in mediating changes in the flux through the glucose-6-phosphate isomerase enzyme [32,60,61]. It was shown that the negative control of the glucose-6-phosphate dehydrogenase on the steady state flux through the glucose-6-phosphate isomerase was mediated by an elevated level of its inhibitor 6-phosphogluconate, which was partly compensated by increased substrate and decreased product levels. Another finding was that in HepG2 cells, oxidative phosphorylation had a significant positive control over the metabolic fluxes in glycolysis. This means that in contrast to primary rat hepatocytes [8], hepatoma cells are not affected by the Pasteur effect. This finding is in line with previous studies that found an increased glycolytic activity in the presence of adequate oxygen levels in liver cancer cells (Warburg effect) [53,54]. The positive control can possibly be ascribed to fewer mitochondria in hepatoma cells [52] or to mitochondrial dysfunction due to mitochondrial DNA mutations [47]. This finding supports approaches that aim at exploiting the Warburg effect for the treatment of tumors [54,55]. It is important to note that the NADPH-demand does not have exclusive control over the rate of NADPH consumption (0.65). Instead, the control is shared with the NADPH supply (0.38). In accordance with previous studies dealing with the control of the consumption of the cofactor ATP in isolated rat hepatocytes [8], it is increasingly becoming clear that also with regard to NADPH the production and the consumption share the control of the NADPH-consuming reactions. The pyruvate dehydrogenase complex was found to have a substantial positive control over the complete TCA cycle. In addition, different control patterns were observed for the first and the last reactions in the TCA cycle. The metabolic influx into the TCA cycle could be enhanced by increasing the cellular NAD and pyruvate levels. However, an increase in pyruvate led to a decreased flux through the reaction mediated by the malic enzyme. This is the reason why ATP consumption has both a positive and negative control over the first and last TCA cycle reactions.

Similarly, glucose-6-phosphate dehydrogenase negatively controls the end of the TCA cycle. The negative control is mainly mediated by an increased NADPH/NADP ratio. The subsequent reaction steps in the TCA cycle are negatively controlled by elevated product levels. The concept of partial flux control proved to be essential for unraveling these control structures. It should be noted that the detailed control structures unraveled in this work had been mainly compared with hepatic control principles obtained from applying top-down approaches in rat hepatocytes [8,32]. To further compare our results with a healthy reference state, it would be interesting to see whether these previously reported control distributions based on finite perturbations can be reproduced using dynamic modeling and *in vivo *metabolite time-series measurements from primary human cells. Moreover, compartmentalization was not accounted for in the dynamic network model. Thus, control principles affected by compartmentalization might differ to some extent from the results obtained in this work, e.g. cytosolic intermediates may have less effects on mitochondrial enzymes and vice versa.

Partial internal response coefficients were determined in order to investigate the reaction steps that are most relevant in counteracting an increase in intermediates in order to return to the steady state [32,62].  Interestingly, in the case of the metabolites in the downstream part of the glycolysis (1,3-bisphospho-glycerate, glycerate-3-phosphate, glycerate-2-phosphate, and phosphoenolpyruvate), an increase in the concentration was almost exclusively counteracted by additional consumption. This possibly suggests that the corresponding enzymes were close to equilibrium [32].

It is envisaged that in the near future, it will be possible to predict the effects of nutrients in the liver at the inter-individual level by coupling metabolic network models to gene regulation and by integrating individual transcriptome and proteome data. Moreover, systems-oriented analyses of hepatic responses to xenobiotics might enable the personalized prognosis of drug actions and/or their persistency.

## Methods

### Experimental Setup and Chemical Analytics

HepG2 cells (ATCC^® ^Number HB-8065™) were incubated at 37°C in 6-well-plates in 5% CO_2 _atmosphere. The cells were cultured in alanyl-glutamine-free William's medium E (PAN Biotech GmbH, Aidenbach, Germany) that was supplemented with penicillin (100 U/mL), streptomycin (100 mg/mL), and Gibco™ Insulin-Transferrin-Selenium (100X) supplement (Invitrogen, Karlsruhe, Germany). No fetal calf serum was added to the medium. The 6-well plates were shaken at 20 rpm throughout the experiment (Shaker DRS-12, ELMI, Riga, Latvia). The number of cells was determined with a Neubauer counting chamber. The intracellular flux map corresponding to this experimental setup was determined previously [6,7]. The main flux was found to be the conversion of glucose to lactate. Thus, for designing an efficient stimulus response experiment, the glucose flux was considered as the most promising candidate for perturbing the central metabolism of the hepatoma cells. However, the cells were grown in a batch culture, and extracellular glucose was provided in excess. Therefore, it was concluded that an extracellular glucose pulse would not yield essential changes, whereas glucose deprivation was expected to trigger a substantial metabolic response. Before depriving the cells of extracellular glucose, they were treated as previously described [6]: The overnight medium was replaced with fresh culture medium, which was then exchanged with glucose-free medium after 2 h of equilibration. Extra- and intracellular samples were collected in triplicate directly before and after the stimulus, as well as 1, 2, 5, 10, 30, 60, 120, and 180 min after glucose deprivation. The sampling approach and the processing of the samples were done as previously described [6].

The concentrations of alanine, serine, glucose, lactate, pyruvate, fumarate, malate, cis-aconitate, isocitrate, and citrate were determined by GC-MS as described before [6,63]. After glucose deprivation, the extracellular glucose concentrations were determined in 10 μl of diluted (1:9 v/v) medium samples, the intracellular glucose concentrations before and after perturbation were determined in 5 and 50 μl of cell extract, respectively. Phosphoenolpyruvate, 3-phosphoglycerate, dihydroxyacetonphosphate, fructose-1,6-bisphosphate, glucose-6-phosphate, 6-phosphogluconate, sedoheptulose-7-phosphate, ribose-5-phosphate, and ribulose-5-phosphate were determined by LC-MS-MS as described by Hofmann et al. [6] with the following modifications: HPLC separation was performed at 20°C on a Synergi Hydro-RP column (150 × 2 mm, 4 μm; Phenomenex, Aschaffenburg, Germany) at a flow rate of 0.2 ml/min. The mobile phases consisted of (A) water with 10 mM tributylamine and 15 mM acetic acid, and (B) methanol. Gradient runs were programmed as follows: 100% A from 0 to 10 min, increase to 20% B to 25 min, remaining at 20% B to 30 min, increase to 35% B to 35 min, remaining at 35% B to 40 min, increase to 60% B to 45 min, increase to 90% B to 48 min remaining at 90% B to 50 min, then equilibrating with 100% A for 13 min. Precursor and product ions used for the quantification of glucose-6-phosphate, 6-phosphogluconate, ribose-5-phosphate, ribulose-5-phosphate, fructose-1,6-bisphosphate, and the internal standard mannitol-1-phosphate were as previously described [6] and for phosphoenolpyruvate: *m/z *167/97, 139; 3-phosphoglycerate: *m/z *185/97, 167; dihydroxyacetonphosphate: *m/z *169/97 and sedoheptulose-7-phosphate: *m/z *289/97, 199.

Nucleotide analysis was performed by reversed phase ion pair high performance liquid chromatography. The HPLC system (Agilent Technologies, Waldbronn, Germany) consisted of an Agilent 1200 series autosampler, an Agilent 1200 series Binary Pump SL, an Agilent 1200 series thermostatted column compartment, and an Agilent 1200 series diode array detector set at 260 and 340 nm. The nucleotides were separated and quantified on an RP-C-18 column that was combined with a guard column (Supelcosil LC-18-T; 15 cm × 4.6 mm, 3 μm packing and Supelguard LC-18-T replacement cartridges, 2 cm; Supelco, Bellefonte, USA) at a flow rate of 1 ml/min. The mobile phases were (i) buffer A (0.1 M KH_2_PO_4_/K_2_HPO_4_, with 4 mM tetrabutylammonium sulfate and 0.5% methanol, ph 6.0) and (ii) solvent B (70% buffer A and 30% methanol, pH 7.2). The following gradient programs were implemented: 100% buffer A from 0 min to 3.5 min, increase to 100% B until 43.5 min, remaining at 100% B until 51 min, decrease to 100% A until 56 min and remaining at 100% A until 66 min.

### Model Reconstruction

A metabolic network model was reconstructed for the identification of hepatic metabolite dynamics. The model was based on a previously published isotopomer model used for the estimation of intracellular fluxes from transient ^13^C-labeling data [7]. The model accounts for 45 balanced compounds that are converted into each other by 49 reactions, including 5 transportation steps. The corresponding metabolic scheme is shown in Figure 1 and the complete reaction stoichiometry is listed in Table 1. The metabolic pathways under consideration contain 3 conserved moieties (c_AMP_+c_ADP_+c_ATP _= const; c_NADP_+c_NADPH _= const; c_NAD_+c_NADH _= const). The model comprises glycolysis (EMP), the pentose-phosphate pathway (PPP), and the tricarboxylic acid (TCA) cycle. In the cataplerotic section, the malic enzyme, which decarboxylates malate to pyruvate, is taken into account. Reduced NADH is regenerated in the lactate dehydrogenase and oxidative phosphorylation reactions. P/O ratios of 2.5 and 1.5 were assumed for NADH and succinate, respectively [64]. No consumption of acetyl-CoA other than through condensation with oxaloacetate by citrate synthase was included [7]; i.e. lipid synthesis was neglected, and, thus, the flux of acetyl-CoA into the tricarboxylic acid cycle may be slightly overvalued in the network model [65]. Based on experimental evidence [7], the metabolic state was assumed to be that of fed hepatic cells. Accordingly, no gluconeogenetic reactions were included. Exchange fluxes with the system boundary took into account glucose and alanine uptake, glycogen storage, the metabolism of glutamate, valine, leucine, and methionine, glycerol and nucleotide synthesis, as well as serine, lactate, and pyruvate excretion. In addition, reactions that represented ATP and NADPH consumption relating to the basal metabolism were included. 31 regulatory effects (21 inhibitions and 10 activations) were found in a literature search [66] and included (cf. Table 5). The network model discriminated 5 extracellular (glucose, lactate, serine, pyruvate, alanine) and 40 intracellular metabolites. The sampling and quenching routine used in this work did not allow discriminating between compartmental concentration differences and, thus, compartmentalization was not accounted for in the dynamic network model. However, it should be noted that the simulated metabolite dynamics in the TCA cycle represent average values integrating cytosolic and mitochondrial network dynamics. The metabolic pathways neither contained dead-end metabolites nor strictly detailed balanced sub-networks [67]. Furthermore, all reactions were consistent with respect to mass conservation and redox state. The stability of the dynamic model was investigated by calculating the eigenvalues of its Jacobian matrix (cf. sub-section Systems-level Analyses). All real parts of the eigenvalues were found to be negative, which means that the system was asymptotically stable. It was important to demonstrate the asymptotic stability of the dynamic model with regard to the envisaged control analysis because in earlier studies it was seen that large-scale dynamic network models tended to be prone to instability [68].

**Table 5:** Activator and inhibitor influences

Reaction Identifier	EC-Number	Activators	Inhibitors
Glucokinase	2.7.1.2		F6P [76]
Glucose-6-phosphate isomerase	5.3.1.9		6PG [44]
6-phosphofructokinase	2.7.1.11	AMP [16]	CIT [77]
Fructose-bisphosphate aldolase	4.1.2.13		ADP, ATP, E4P, F6P, G1P, G6P, RIBO5P [78]
Triose-phosphate isomerase	5.3.1.1		ATP [79]
Glyceraldehyde-3-phosphate dehydrogenase	1.2.1.12		ADP, ATP [80]
Phosphoglycerate kinase	2.7.2.3		AMP [81]
Pyruvate kinase	2.7.1.40	G6P, F6P, G1P [82], F16P [83]	ALA [84]
Glucose-6-phosphate dehydrogenase	1.1.1.49		ATP [58]
Phosphoglucomutase	5.4.2.2		f16p [85]
UTP-glucose-1-phosphate uridylyltransferase	2.7.7.9		AMP [16]
Alpha-ketoglutarate dehydrogenase	1.2.4.2	ADP [86]	ATP [86]
Valine, isoleucine, methionine metabolism	-	NAD, AKG [87]	GLU, NADH [87]
Isocitrate dehydrogenase	1.1.1.41	ADP [88]	
Pyruvate dehydrogenase	1.2.4.1	AMP [89]	

Regulatory influences and corresponding literature references. The modulator effects were included in the dynamic network model. In addition to these regulatory influences, the dynamic model did also account for substrate and product effects.

### Model Simulation and Parameterization

The following set of metabolite mass balances was set up to describe the time-dependent behavior of the metabolic system presented above:(1)

**N **denotes the stoichiometric matrix and **r **the rate vector. (**c**^0^) is a square diagonal matrix with reference concentrations on its main diagonal;  denotes the normalized metabolite concentration vector.

The ordinary differential equations (ODEs) were reformulated as differential algebraic equations (DAEs) to improve both the performance and stability of the numerical integrations, i.e. the conservation relations were solved algebraically. The DAE system was simulated with the linearly implicit differential algebraic solver LIMEX [69]. On average, one simulation run took 0.2 seconds (Intel^® ^Core2™ Quad CPU, 2.66 GHz, 4 GB RAM).

Canonical linear-logarithmic (linlog) kinetics were applied for approximating the reaction rates in equation (1) [70-72]. The linlog formalism has been used for modeling *in vivo *kinetics and metabolic redesign [70]. Linlog kinetics were shown to have a good approximation quality and to need only relatively few parameters to be identified [22,38,39]. In linlog kinetics, all rate equations share a standardized mathematical format in which influences of metabolite and effector levels on reaction rates are taken into consideration by adding up logarithmic concentration terms. The standardized format is advantageous if not all kinetic mechanisms are known in detail, which is often the case even for well-studied pathways of the central carbon metabolism [2,73]. The matrix notation of the linlog rate equation is given by [70](2)

in which **J**^0 ^is the reference steady state flux distribution,  is a diagonal matrix containing relative enzyme levels, and **i **is a vector of ones.  is a matrix whose entries are scaled elasticity coefficients ϵ_ij _that describe the local effect of an infinitesimal change in concentration j on the rate of reaction i, i.e.(3)

Assuming constant enzyme levels, equation (2) can be reduced to(4)

Parameterizing the kinetic model requires the specification of a reference steady state, i.e. **J**^0 ^and **c**^0^, and the corresponding kinetic parameters, i.e. . Therefore, a two-step approach was applied. In a previous study, **J**^0 ^was estimated from transient ^13^C-labeling data [6,7]. In the present study, **c**^0 ^and  are determined from stationary and non-stationary metabolite measurements. Each rate equation was assumed to be dependent on its substrate and product levels. In some instances additional effectors were taken into account; for details see Table 5. Altogether, 174 scaled elasticities had to be estimated. The matrix of scaled elasticity values is listed in Additional file 5. Furthermore, 42 reference intermediate levels had to be identified (42 balanced compounds + 3 conserved moieties). The corresponding experimental data were available for 30 of these. This means that 216 unknown parameters had to be specified in order to run a simulation.

At the outset of a simulation run, all intracellular metabolite levels  were set to 1.0. The initial values for the extracellular metabolites were determined by(5)

where  denotes the extracellular metabolite levels immediately after the perturbation. The unknown elasticity coefficients and reference concentrations were identified by minimizing the differences between *in silico *model simulations and *in vivo *measurement data: The variance-weighted sum of squared residuals χ^2 ^between experimentally observed and simulated metabolite data, **c**^m ^and **c**^s^, was minimized according to(6)

in which Σ_m _is a diagonal matrix containing the measurement variances. An evolution strategy was applied for parameter fine-tuning that included a self adapting mutation operator [74,75]. To enable a thorough exploration of the search space, the optimization runs were restarted after 100,000 evaluations of equation (6) using the best parameters currently available as starting values in the following iteration. Altogether, more than six million simulation runs were performed.

No confidence limits were calculated for the estimated parameters. For parameter estimation, however, a multi-start optimization approach was taken and the distribution of control was consistently determined from the parameters estimated and, thus, was considered to be reliable. It is worth noting that in an exploratory study, Nikerel et al. investigated the identification of kinetic parameters in a dynamic model based on linlog kinetics, and found that the underlying control structures were inherently robust against non-identifiable elasticities [22]. Moreover, due to the substantial nonlinearities of the dynamic network model, methods based on linearization, like e.g. the inversion of the Fisher information matrix (FIM), are inadequate, whereas Monte-Carlo-based approaches would be most suitable to determine the confidence ellipsoids. However, Monte-Carlo methods are computationally too demanding for the outlined model complexity.

### Systems-Level Analyses

In this study, the local stability of the biochemical system was investigated by analyzing the eigenvalues of its Jacobian matrix **J **given by(7)

where **N**_R _and **L **are the reduced stoichiometric matrix and link matrix, respectively [30]. A steady state is asymptotically stable if all real parts of the eigenvalues of the Jacobian matrix J are negative.

A metabolic control analysis was carried out to assess systemic steady state properties. In this context, the concentration control coefficient  determines the relative effect a changing enzyme level j has on steady state concentration i, i.e.(8)

Similarly, flux control coefficient  describes the relative effect changing enzyme level j has on steady state flux i, i.e.(9)

By applying the summation and connectivity theorems, the concentration and flux control coefficients can be calculated from the estimated elasticities and steady state concentrations [30](10)

and(11)

The control that one enzyme exerts over another is mediated by changes in the levels of intermediates. Enzymatic reaction rates respond to changes in substrate, product, and effector levels. The concept of a partial flux control coefficient allows the quantification of the fractions of the flux change in relation to changes occurring in individual intermediates [32,60,61]. Partial flux control coefficients can be obtained by partitioning flux control coefficients. In accordance with equation (11), the flux control coefficient , which quantifies the change in steady state flux i with respect to changes in enzyme j, is given by(12)

The individual summands  are termed partial flux control coefficients. They partition the flux control coefficient through the changes in each intermediate, i.e.  quantifies what fraction of the change in the flux through enzyme i can be attributed to the change in metabolite x. Initially, partial flux control coefficients were divided by the total flux control coefficient and referred to as conditional elasticities [60] and partitioned regulatory coefficients [61]. However, Ainscow and Brand pointed out that the normalization precludes easy comparison between partitioned terms of different flux control coefficients [32]. Therefore, partial flux control coefficients were not scaled in the present study.

A stable system that operates at steady state will counteract changes in intermediate levels and eventually return to its original steady state [32]. Elevated intermediate concentrations may be counteracted by a decrease in production and/or elevated consumption of these intermediates. By quantifying these effects, the partial internal response coefficients  allow the assessment of their importance in restoring the steady state [32,62], i.e.(13)

According to the connectivity theorem the sum of the partial internal response coefficients for each intermediate is -1. For a given enzyme j and metabolite i, the internal response coefficient  is identical to the partial flux control coefficient of the enzyme over itself [32]. Graphically oriented network set-up, automated generation of the DAE system, and the quantification of metabolic control were performed with the Insilico Discovery software (Insilico Biotechnology AG, Stuttgart, Germany).

## Abbreviations

PPP: pentose-phosphate pathway; TCA: tricarboxylic acid; EMP: Embden-Meyerhof-Parnas; VLDL: very-low-density lipoproteins; HPLC: high performance liquid chromatography; GC-MS: gas chromatography-mass spectrometry; LC-MS-MS: liquid chromatography-mass spectrometry-mass spectrometry; ODE: ordinary differential equation; DAE: differential algebraic equation; ROS: reactive oxygen species; GLC: glucose; G6P: glucose-6-phosphate; G1P: glucose-1-phosphate; F6P: fructose-6-phosphate; F16P: fructose-1,6-bisphosphate; DHAP: dihydroxyacetone phosphate; GAP: glyceraldehyde 3-phosphate; 13PG: 1,3-bisphospho-glycerate; G3P: 3-phosphoglycerate; G2P: 2-phosphoglycerate; PEP: phosphoenolpyruvate; PYR: pyruvate; SER: serine; LAC: lactate; ALA: alanine; GL6P: 6-phospho-glucono-1,5-lactone; 6PG: 6-phospho-gluconate; RIBU5P: ribulose 5-phosphate; RIBO5P: ribose 5-phosphate; XYL5P: xylulose 5-phosphate; S7P: sedoheptulose 7-phosphate; E4P: erythrose 4-phosphate; ACCOA: acetyl-CoA; CIT: citrate; CISAC: cis-aconitate; ISOCIT: isocitrate; AKG: alpha-ketoglutarate; SUCCOA: succinyl-CoA; SUC: succinate; FUM: fumarate; MAL: malate; OAC: oxaloacetate; ATP: adenosintriphosphate; ADP: adenosindiphosphate; AMP: adenosinmonophosphate; NADP(H): nicotinamide adenine dinucleotide phosphate; NAD(H): nicotinamide adenine dinucleotide; r1: glucokinase; r2: glucose-6-phosphate isomerase; r3: phosphofructokinase; r4: fructose-bisphosphate aldolase; r5: triose-phosphate isomerase; r6: glyceraldehyde-3-phosphate dehydrogenase; r7: phosphoglycerate kinase; r8: phosphoglycerate mutase; r9: pyruvate kinase; r10: glucose-6-phosphate dehydrogenase; r11: 6-phosphogluconolactonase; r12: phosphogluconate dehydrogenase; r13: ribose-5-phosphate isomerase; r14: ribulose-phosphate 3-epimerase; r15: transketolase; r16: transketolase; r17: phosphopyruvate hydratase; r18: lactate dehydrogenase; r19: adenosinetriphosphatase; r20: alanine transaminase; r21: phosphoglucomutase; r22: NADPH consumption; r23: glycogen synthesis; r24: transaldolase; r25: adenylate kinase; r26: glycerol formation; r27: nucleotide synthesis; r28: serine synthesis; r29: citrate synthase; r30: aconitate hydratase; r31: aconitate hydratase; r32: isocitrate dehydrogenase; r33: succinate-CoA ligase; r34: fumarate hydratase; r35: malate dehydrogenase; r36: pyruvate dehydrogenase complex; r37: alpha-ketoglutarate dehydrogenase complex; r38: pyruvate synthesis; r39: valine leucine isoleucine metabolism; r40: succinate dehydrogenase; r41: oxidative phosphorylation; r42: alpha-ketoglutarate synthesis; r43: malic enzyme; r44: glutamate dehydrogenase; r45: lactate transport; r46: pyruvate transport; r47: glucose transport (GLUT2); r48: alanine transport; r49: serine transport;

## Authors' contributions

KM^a ^was involved in the design and execution of the perturbation experiment, he also performed the model identification and systems-level analyses, conceived the study, and drafted the manuscript. UH contributed to the design of the experimental setup, coordinated the chemical analyses and participated in the design of the study. MR and KM^b ^conceived the study and contributed to its design. KM^b ^coordinated the experimental design, model set-up and analysis, and co-drafted the manuscript. All authors read and approved the final manuscript.

## Supplementary Material

Additional file 1 **Reference extracellular and intracellular metabolite levels**. The subscripts 'ex' and 'in' denote extracellular and intracellular metabolites, respectively. Intracellular and extracellular values are given in mmol/l_cv _and mmol/l_well_. Click here for file

Additional file 2 Flux control coefficients Click here for file

Additional file 3 Concentration control coefficients Click here for file

Additional file 4 **Partial internal response coefficients**. According to the connectivity theorem, the sum of the partial internal response coefficients for each intermediate is -1. Perturbations in moiety-conserved sets cannot be counteracted in order to reach the previous steady state, which is the reason why no partial response coefficients are listed for ATP, ADP, AMP, NADP, NADPH, NAD, and NADH. Click here for file

Additional file 5 **Elasticity matrix**. The scaled elasticities are based on metabolite time-series data. Only non-zero entries are shown. The subscript 'ex' denotes extracellular metabolites. Click here for file
